# Proton beam therapy induces protective immunity via HMGB1-dependent signaling

**DOI:** 10.3389/fpubh.2025.1686678

**Published:** 2026-01-20

**Authors:** Jialing Wen, Xuanzhang Tu, Wangcai Ren, Qiaojuan Wang, Yue Wang, Gang Guo, Kensuke Osada, Takashi Shimokawa, Akihisa Takahashi, Nakako Izumi Nakajima, Shenke Zhang, Wenchao Gu, Yang Li, Chen Li, Li Sui, Liqiu Ma

**Affiliations:** 1 Department of Nuclear Physics, China Institute of Atomic Energy, Beijing, China; 2 College of Physics and Optoelectronic Engineering, Shenzhen University, Shenzhen, China; 3 Institute for Quantum Medical Science, National Institutes for Quantum Science and Technology (QST), Chiba, Japan; 4 Gunma University Heavy Ion Medical Center, Maebashi, Gunma, Japan; 5 QST Hospital, National Institutes for Quantum Science and Technology (QST), Chiba, Japan; 6 Department of Gastroenterology and Hepatology, The First Affiliated Hospital of Zhengzhou University, Zhengzhou University, Zhengzhou, Henan, China; 7 Department of Artificial Intelligence Medicine, Graduate School of Medicine, Chiba University, Chiba, Japan; 8 Department of Radiation Oncology, Harbin Medical University Cancer Hospital, Harbin, China; 9 China CDC Key Laboratory of Radiological Protection and Nuclear Emergency, National Institute for Radiological Protection, Chinese Center for Disease Control and Prevention, Beijing, China

**Keywords:** calreticulin, high mobility group box 1, immunogenic cell death, proton beam radiation, tumor colonization rejection

## Abstract

Proton beam therapy is widely regarded for its cost-effectiveness, precision, and protection of normal tissues. Emerging evidence shows that conventional radiotherapy can inhibit primary tumors and promote immunogenicity of distant tumors, possibly via damage-associated molecular patterns (DAMPs) like calreticulin (CRT) and high mobility group box 1 (HMGB1) released during immunogenic cell death (ICD). However, the role of proton beams in inducing DAMPs and enhancing immunogenicity remains unclear. This study aimed to investigate the effects of proton beam-induced DAMPs on the colonization of distal tumors. In this study, *in vitro* cell irradiation experiments were conducted to identify the optimal proton beam dose for enhancing DAMPs expression in mouse colon carcinoma Colon-26 cells. Based on the optimal proton dose determined *in vitro*, a tumor-bearing mouse model was employed to evaluate its efficacy in inhibiting distal tumor colonization. To explore the mechanisms behind the anti-tumor effects, shRNA targeting DAMPs-related immunogenic molecules was applied to assess the immune response. *In vitro* findings indicated high-dose proton irradiation markedly induces HMGB1 release yet exerts no significant effect on CRT membrane exposure. Following high-dose proton beam irradiation, tumor cells transfected with shRNA exhibited a significant reduction in CRT and HMGB1 expression compared with the con-shRNA-irradiated control group. *In vivo* experiments demonstrated that HMGB1 knockdown reduced distal-tumor rejection by 60%, whereas CRT knockdown reduced it by only 20%, indicating that HMGB1 release may dominate proton-induced ICD. Our research results indicated that high-dose proton irradiation trigger the rejection of distal tumor colonization through a signaling pathway that depends on HMGB1. This research advanced our understanding of proton beam therapy immunological mechanisms and offered insights for improving tumor treatment outcomes.

## 1 Introduction

Cancer seriously endangers human health and has become the second leading cause of death after cardiovascular disease (1). Preventing cancer has thus become one of the important public health challenges in the 21st century. Although cancer research and treatment have made significant advancements in recent years, the associated mortality rate continues to rise, with cancer metastasis being one of the primary contributing factors (2, 3). Cancer metastasis refers to the process by which tumor cells detach from the primary site, disseminate via the bloodstream or lymphatic system to distal organs, and establish secondary tumors at these new locations (4, 5). Metastatic tumors are typically more aggressive than primary tumors and exhibit greater resistance to conventional treatment modalities (6). Consequently, cancer metastasis has emerged as a critical determinant influencing patient survival rates and quality of life, underscoring the urgent need to develop efficacious treatment strategies for the benefit of cancer patients.

Proton beam therapy is increasingly recognized as a promising treatment modality within the realm of tumor radiotherapy, owing to its notable advantages (7, 8). These include its superior cost-effectiveness, the precise and conformal irradiation achievable to tumor target areas, and the effective preservation of adjacent normal tissues (8–10). Proton beam therapy offers better clinical advantages than traditional photon therapy in treating cancer patients, including better survival outcomes and lower radiation toxicity (11–14). Notably, it is confirmed that proton beam irradiation significantly reduces the radiation dose absorbed by immune cells in the blood, lymph nodes, spleen, and bone marrow during treatment compared with conventional X-ray therapy, thus providing enhanced protection for these critical immune cells (15). Preserving these immune reservoirs maintains the pool of effector and memory T cells, which are indispensable for systemic anti-tumor immunity; consequently, proton beam therapy is expected to potentiate endogenous immune surveillance and improve therapeutic outcomes beyond pure physical dose sparing (16). Additionally, several studies indicate that proton beams can induce more robust immunogenic cell death (ICD) in tumor cells, thereby releasing damage-associated molecular patterns (DAMPs). These DAMPs, such as calreticulin (CRT) and high mobility group box 1(HMGB1), bind to dendritic cells (DCs), subsequently activating T-cell-mediated cytotoxicity against distal tumors (17–21).

Despite the remarkable potential of proton beam therapy in clinical settings, the mechanism by which it triggers anti-tumor immunity is only partially understood. Hence, there is a pressing need for in-depth studies to investigate the potential benefits of proton beam therapy in activating anti-tumor immune responses and to enhance the efficacy of proton beam therapy. Thus, we employed a DAMPs knockdown tumor-bearing mouse model to evaluate the inhibitory effect of proton beam therapy on distal tumors and to explore the underlying mechanisms related to DAMPs. Our research could provide novel strategies and methods for cancer treatment, optimize the clinical application of proton beam therapy, and possibly bring about new breakthroughs in cancer therapy.

## 2 Material and method

### 2.1 Mice and cell lines

Six-week-old female BALB/c mice were obtained from China MDL Co., Ltd. (Langfang, China). All experiments were conducted in accordance with the recommendations (approval number: MDL2024-12-22-1, MDL2025-03-03-020). Mice were housed under specific-pathogen-free (SPF) conditions with ad libitum access to food and water. For euthanasia, mice were sacrificed by cervical dislocation without prior sedation, following approved protocols to minimize suffering. The murine colon carcinoma cell line Colon-26 was purchased from Shanghai Jinyuan Co., Ltd. (Shanghai, China). Colon-26 cells were cultured in RPMI 1640 (HyClone, Logan, UT, USA). Stably transfected Colon-26-shCRT and Colon-26-shHMGB1 cells were purchased from Oligo Bioscience Co., Ltd. (Beijing, China) and cultured according to the supplier’s instructions. Complete medium was supplemented with 10% fetal bovine serum (HyClone, Logan, UT, USA) and 1% penicillin–streptomycin (HyClone, Logan, UT, USA). All the cells were maintained at 37 °C in an incubator containing 5% CO_2_.

### 2.2 Cell irradiation

Proton irradiation experiment was conducted at the China Institute of Atomic Energy (CIAE). Forty-eight hours prior to irradiation, Colon-26 cells were inoculated into T25 tissue culture flasks, which were irradiated by proton (Energy: 90 MeV; LET: 0.985 KeV/μm; Dose rate: 3 Gy/min). For clonogenic assays, the cells were irradiated with 2, 3, 5, 6, 10 or 12 Gy and immediately seeded into 6 cm culture dishes after irradiation. For the purpose of other experiments, the cells were irradiated with 3, 6, 12 Gy and processed 24 or 48 h after irradiation. Notably, the experiments involving shRNA treatment were conducted using 12 Gy.

### 2.3 Clonogenicity assays

Post-irradiation, the cells were immediately harvested and counted as individual cells. Subsequently, the cells were plated into 6 cm culture dishes and incubated in a culture incubator maintained at 37 °C with 5% CO_2_ and saturated humidity for 10 days. Three parallel tests were performed for each dose group to ensure reproducibility. Following Giemsa staining, colonies comprising more than 50 cells were counted. The surviving fraction (SF) was calculated according to Equation 1:


$$\text{SF}=\frac{\text{No. of colonies formed after irradiation with a certain dose}}{\text{No. of seeded cells}\times \text{PE}}$$ (1)

PE is the fraction of colonies from cells not exposed to the treatment.

Survival data after a radiation dose were fitted by a weighted, stratified, linear regression according to the linear-quadratic Equation 2:


$$S=e^{-(\mathrm{\alpha D}+\beta D^{2})}$$ (2)

S is the cell survival rate; *α* represents the linear effect; *β* represents the square effect; D is the dose (22).

### 2.4 Flow cytometry analysis

For the expression of CRT on the cell surface *in vitro* experiments, the cells were gently lifted using a cell scraper and subsequently collected. Collected cells were resuspended in 400 μL of FACS Buffer (00-8333, Thermo, USA) and divided into two aliquots: one for anti-CRT (1:50, ab196158, Abcam, USA) staining and the other for isotype antibody (1:50, ab199091, Abcam, USA) staining (*n* = 3 samples per group). The antibody-treated cells were incubated on ice for 30 min and then washed twice with FACS buffer. Cell viability was assessed by adding 5 μL of 7-Aminoactinomycin D (7-AAD, 00-6993-50, Thermo, USA) to each tube. After incubation on ice in the dark for 15 min, CRT on the cell surface was detected with an Attune™ Acoustic Focusing Cytometer (Thermo, USA). As negative control, isotype-matched labeled were used in all experiments. For the determination of T lymphocytes in the spleen of mice, the spleens were collected and erythrocytes were lysed with red blood cell lysis buffer (#07800, Stemcell Technologies, Vancouver, Canada) after mice were sacrificed by cervical dislocation. Single-cell suspensions were generated by gently forcing the cells through a disposable cell strainer (70 μm) into cold PBS. Cells were centrifuged at 1000 rpm for 3 minutes, after which the supernatant was discarded and the spleen cells were collected. Subsequently, 1 × 10^5^ splenocytes were resuspended in a staining buffer containing specific antibodies that were prepared by diluting three specific antibodies in the FACS Buffer at a ratio of 1:50 (APC anti-CD3, 100236; PE anti-CD4, 100408; FITC anti-CD8a 100706, Biolegend, USA). Flow cytometry was conducted using a BENM DIAG BeamCyte-1026 flow cytometer (Suzhou, China).

### 2.5 ELISA

HMGB1 expression in the culture supernatants of cancer cells was measured via a competition ELISA kit (H257-1-2, Mouse HMGB1 ELISA Kit, NJJC Bioscience, Nanjing, China) according to the manufacturer’s instructions (*n* = 3 serum samples per group). HMGB1 and Interferon-gamma (IFN-γ) in the mouse serum samples were measured by using HMGB1 kit (H257-1-2, Mouse HMGB1 ELISA Kit, NJJC Bioscience, Nanjing, China) and IFN-γ kit (#EK0375, Boster Biological Technology, Wuhan, China) according to the manufacturer’s instructions.

### 2.6 Vaccine mouse model

5 × 10^5^ Colon-26 cells in 20 μL PBS were injected into the left hind limb of syngeneic BALB/c mice 24 h after *in vitro* proton irradiation (3, 6 or 12 Gy). Additionally, to investigate the inhibitory effects of DAMPs on distal tumors, 5× 10^5^ Colon-26 cells stably knocked out for CRT and HMGB1 via shRNA were transplanted into the left hind limb of mice 24 h after 12 Gy proton irradiation. Seven days later, 1× 10^5^ untreated tumor cells of the same kind were injected into the right hind limb of the mice (*n* = 5 per group). The colonization characteristics of distal tumors were judged by tumor formation. Tumor diameter was measured three times per week after irradiation. The tumor volume was calculated according to the following formula: (a × b × c × *π*)/6. After four weeks, the spleen, lung, and tumor tissue were extracted from the mice.

### 2.7 Statistical analysis

All statistical analyses were conducted using GraphPad Prism software (version 10.1.2, San Diego, CA, USA). Prior to analysis, data normality was assessed using the Shapiro–Wilk and Kolmogorov–Smirnov tests. Based on the normality results, either parametric or nonparametric tests were chosen. Student’s t-test (parametric), Mann–Whitney test (nonparametric), one-way ANOVA (parametric) with Tukey’s *post hoc* test, Kruskal–Wallis test (nonparametric) with Dunn’s post hoc test, Welch’s t-test (parametric; for unequal variances), and Brown-Forsythe and Welch ANOVA with Dunnett’s T3 post hoc test (parametric; for unequal variances) were employed to evaluate the significance of differences. When mean values were plotted, error bars indicated the standard error of the mean. Differences were considered statistically significant at *p* < 0.05.

## 3 Result

### 3.1 Colony formation assay

**Figure 1:**
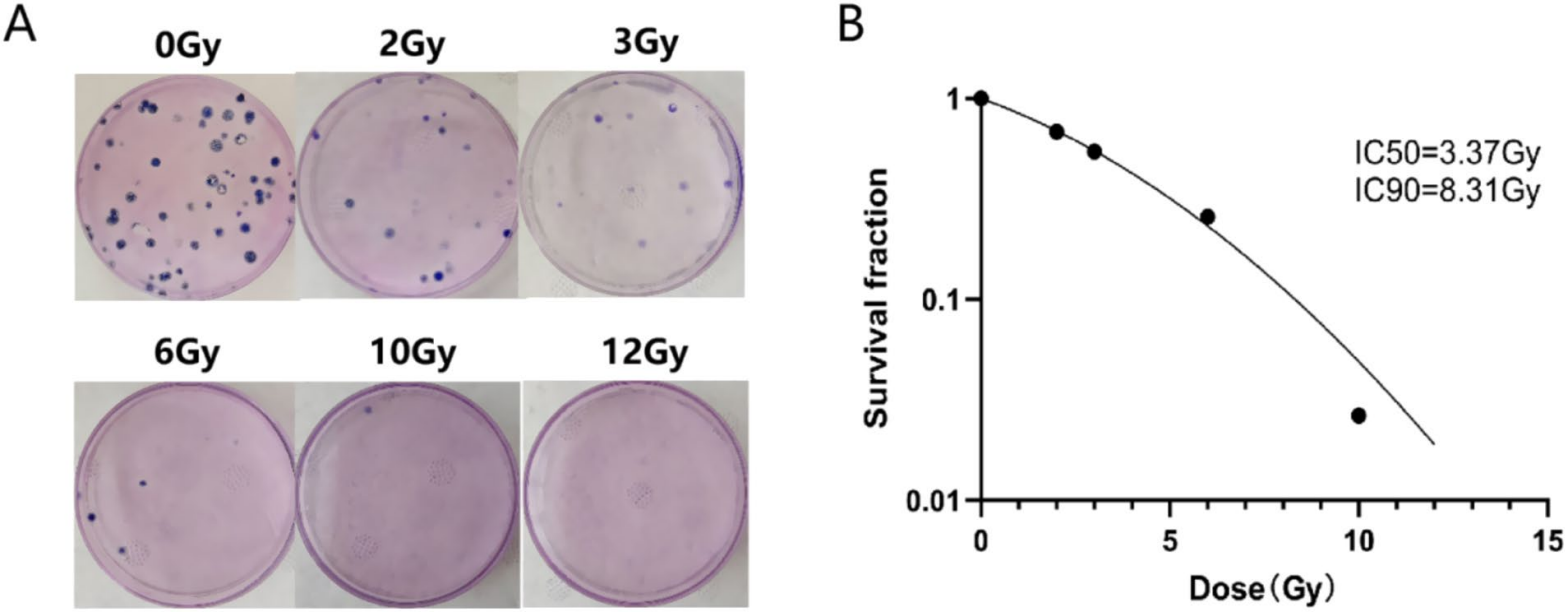
Colony formation assay. **(A)** typical pictures of cell colonies. **(B)** Clonogenic survival curves of Colon-26 cells after proton irradiation at doses of 2, 3, 6, 10, 12 Gy.

To evaluate the cytotoxic effects of proton irradiation on mouse colon carcinoma Colon-26 cells, colony formation assays were performed. As depicted in Figure 1A, the clonogenic survival fraction of Colon-26 cells exhibited a dose-dependent decrease. Figure 1B illustrated the cell survival curve fitted using the linear-quadratic (LQ) model, which further comfirmed the dose-dependent reduction in cell viability. Notably, the semi-lethal dose (LD50) for Colon-26 cells was determined to be 3.37 Gy, while the dose required for 90% lethality (LD90) was 8.31 Gy. Based on these survival data, doses of 3 Gy, 6 Gy, and 12 Gy were selected for subsequent experiments. Additionally, 3 Gy was chosen as it eradicated approximately 50% of cells, 6 Gy was found to eliminate around 80% of cells, and 12 Gy was capable of almost completely abolishing cell viability. This selection allowed for a comprehensive assessment of the effects of different radiation doses on tumor growth and cell survival.

### 3.2 High-dose proton irradiation induces HMGB1 expression while CRT remains unaffected

**Figure 2:**
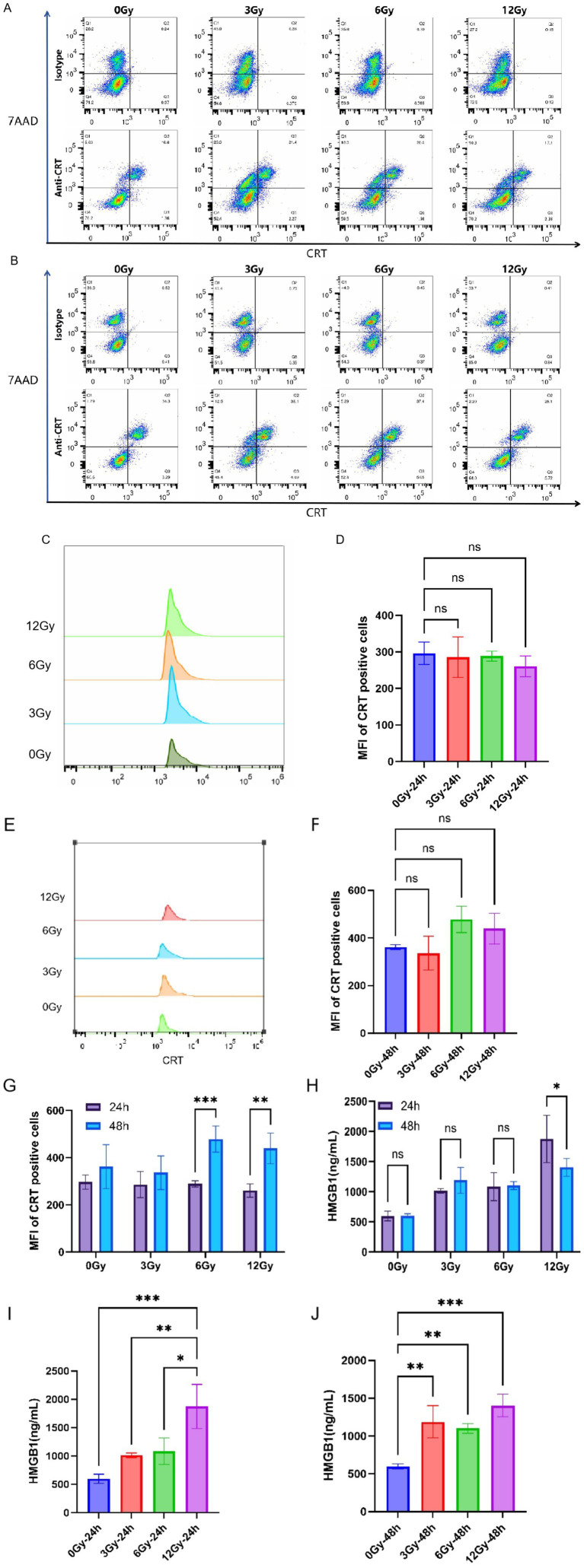
The expression of proton-induced CRT and HMGB1. **(A,C,D)** Flow cytometry analysis of CRT at 24 h post-proton irradiation. **(B,E,F)** Flow cytometry analysis of CRT at 48 h post-proton irradiation. **(G)** The temporal characteristics of proton-induced CRT expression. **(H–J)** The expression of HMGB1 in the supernatant of cell culture medium 24 **(I)** and 48 **(J)** hours after proton irradiation. **(H)** The temporal characteristics of proton-induced HMGB1 expression. MFI means median fluorescence intensity. * means *p* < 0.05, ** means *p* < 0.01,*** means *p* < 0.001, ns means not significant.

As one of the key molecules in anti-cancer immunity, CRT expression is linked to anti-tumor immunity. As an endoplasmic reticulum (ER) stress marker, it promotes DCs antigen presentation and T-cell-mediated immune responses, enhancing tumor cell clearance. Although several stimuli can evoke this translocation, its regulation under proton irradiation remains undefined. Therefore, the cells were subjected to proton irradiation at physical doses of 3, 6, and 12 Gy. CRT exposure was subsequently assessed via flow cytometry at 24 and 48 h post-irradiation (Figures 2A,B). Our results indicated that after Colon-26 cells were exposed to different doses of proton irradiation (3, 6, 12 Gy), there was no significant difference in the expression level of CRT on the cell surface (7-AAD -, CRT +) compared to the non-irradiated group (Figures 2C–F). In addition, the 6 Gy and 12 Gy groups exhibited a significant increase in surface CRT median fluorescence intensity (MFI) at 48 h compared with 24 h (*p* < 0.05), whereas no significant time-dependent change was observed in the 0 Gy or 3 Gy groups (Figure 2G).

HMGB1 is also an important DAMPs and has been confirmed to be closely related to RT-induced anti-tumor immunity. To clarify the effects of different proton doses on HMGB1 release from Colon-26 cells, we assessed HMGB1 expression in the cultured medium supernatant after proton irradiation, and the results were shown in Figures 2I,J. The results demonstrated that, compared to the unirradiated (0 Gy) and low-to-moderate-dose groups (3 Gy and 6 Gy), HMGB1 expression in the culture medium supernatant was significantly elevated in the high dose proton-irradiated group (12 Gy) at 24 h post-irradiation (Figure 2I). At 48 h, proton irradiation markedly increased HMGB1 expression in Colon-26 cells relative to the unirradiated group, yet no significant differences were observed among the various proton-dose groups (Figure 2J). Additionally, at the same dose (except for 12 Gy), no significant difference was observed in HMGB1 expression between 48 and 24 h (Figure 2H).

### 3.3 Proton beams induce immune response in the vaccine mouse models

**Figure 3:**
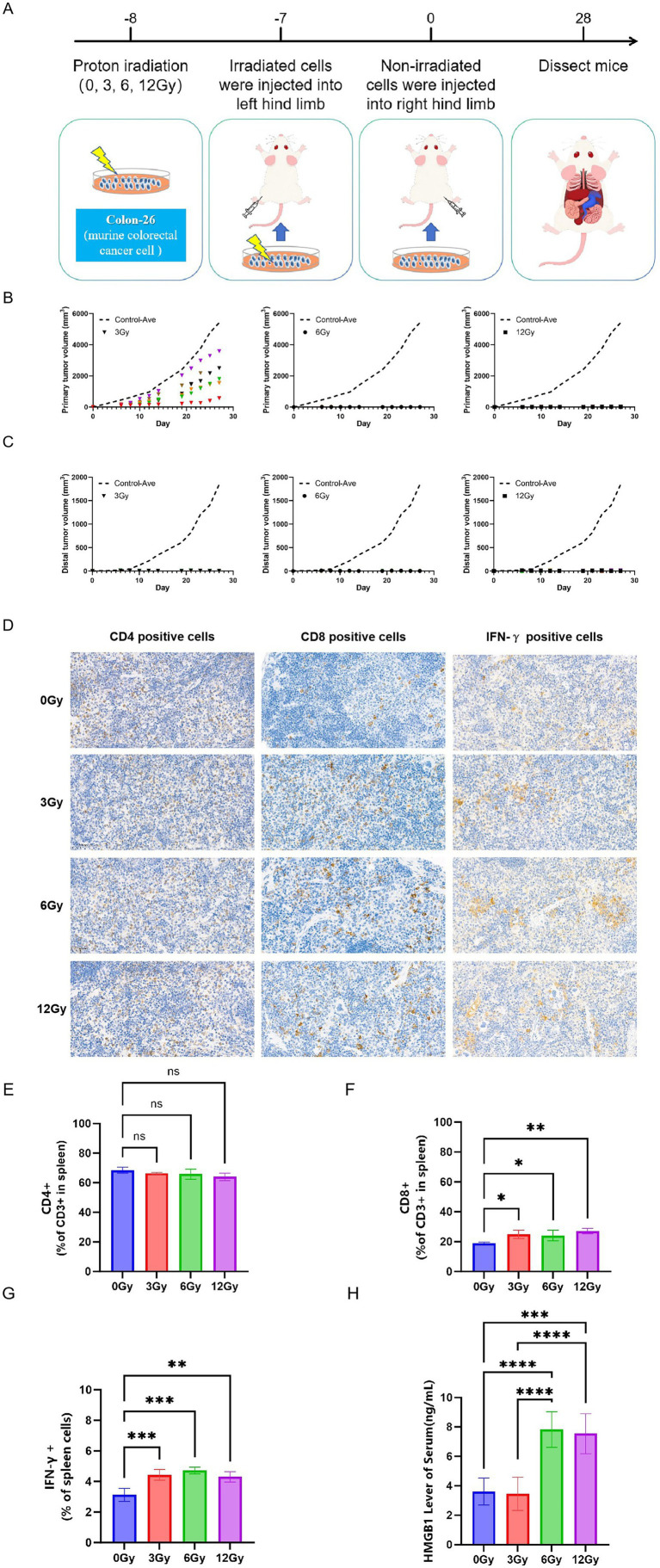
Proton radiation stimulates immunogenic cell death. **(A)** Experimental schedule for proton-induced ICD evaluation. Primary tumor growth **(B)** and distal tumor growth **(C)** after the transplantation of different doses (3, 6, 12 Gy) proton-irradiated cells. Four weeks after tumor cell transplantation, spleens were harvested and analysed: immunohistochemical staining of T lymphocytes **(D)** and IFN-*γ* **(G)** infiltration in the spleen, and quantification by flow cytometry **(E,F)**. **(H)** The expression of HMGB1 in serum after the transplantation of proton-irradiated cells. * means *p* < 0.05, ** means *p* < 0.01,*** means *p* < 0.001, **** means *p* < 0.0001, ns means not significant.

In order to determine the ability of different doses of proton beams to render tumor cells immunogenic *in vivo*, we established a vaccination-challenge model and systematically assessed whether proton-treated tumor cells can elicit protective anti-tumor immunity. We exposed Colon-26 to proton radiation and then injected the cells into the left hind limb of immunocompetent BALB/c mice. The mice were then rechallenged with live tumor cells injected into the opposite limb 7 days later (Figure 3A). Our results showed that low dose (3 Gy) proton-irradiated cells remained viable and proliferated at the primary site but failed to establish tumor at the rechallenged site, suggesting 3 Gy proton beams could not suppress primary tumor growth but could induce distal tumor colonization rejection. In contrast, moderate and high dose (6 and 12 Gy) proton-irradiated cells failed to form tumors at the primary site, and also prevented tumor colonization at a distal site when injected *in vivo* (Figures 3B,C). Subsequently, the profile of immune factors and cells in the spleen was assessed (Figure 3D). No significant difference in CD4 + T cell expression was found between the proton-irradiated vaccine and control groups. However, the results demonstrated a significant increase in splenic CD8 + T cells in the proton-irradiated group compared to the control group (Figures 3E,F). And proton irradiation increased the number of IFN-*γ* positive cells in the spleen compared to the unirradiated group (Figure 3G). Additionally, we quantified serum HMGB1 levels and observed, remarkably, that mice vaccinated with proton-irradiated tumor cells exhibited a significant elevation in HMGB1 compared with the control group (Figure 3H). However, surface CRT could not be assessed because the distal tumors failed to develop, thus precluding acquisition of tumor tissue for CRT analysis.

### 3.4 The gene silencing of DAMPs prevents radiation from altering its expression

**Figure 4:**
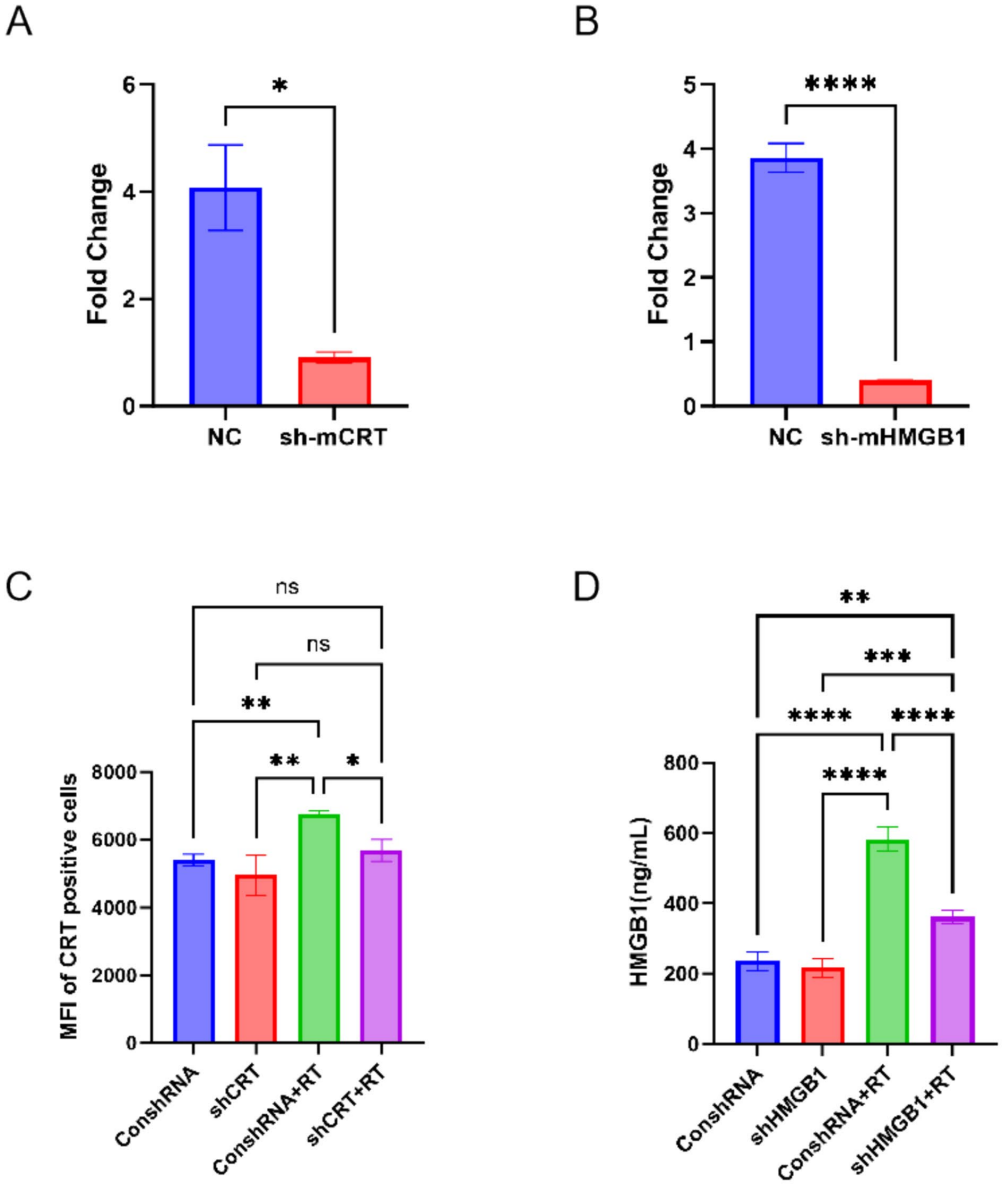
Verification of silencing of shCRT and shHMGB1 cell lines by proton irradiation. The mRNA expression level of CRT **(A)** and HMGB1 **(B)** after corresponding gene silencing. Surface expression of CRT **(C)** and secreted HMGB1 concentration **(D)** in CRT- or HMGB1-knockdown Colon-26 stable cell lines were measured after irradiation by flow cytometry and ELISA, respectively. * means *p* < 0.05, ** means *p* < 0.01,*** means *p* < 0.001, **** means *p* < 0.0001, ns means not significant.

To clarify the roles of CRT and HMGB1 in suppressing distal tumors, we designed and synthesized shRNA sequences specific for CRT and HMGB1 based on their gene sequences in Table 1. These shRNAs were cloned into lentiviral vectors to stably transfect Colon-26 cells, generating specific cell lines: Colon-26-shCRT and Colon-26-shHMGB1. By knocking down CRT and HMGB1 expression, we explored their functions in proton radiation-induced ICD. The mRNA expression levels of CRT and HMGB1 in Colon-26-shCRT and Colon-26-shHMGB1 cells are shown in Figures 4A,B, respectively. CRT-targeting shRNA reduced CRT mRNA by 77.64% compared to the control, while HMGB1-targeting shRNA decreased HMGB1 mRNA by 89.54%. Consequently, Colon-26-shCRT and Colon-26-shHMGB1 were selected for further experiments.

**Table 1:** Knock down the gene target information.

ID	Target sequence information
sh-CRT	GCATGGAGACTCAGAATATAA
sh-HMGB1	TGACAAGGCTCGTTATGAAAG

Our previous experiment indicated that proton irradiation upregulated HMGB1, while evoking no detectable alteration in cell-surface CRT levels. To check if the stably knocked down Colon-26 cell lines still kept low CRT and HMGB1 expression post-irradiation, we measured their expression levels 24 h after irradiation. In Colon-26-shCRT cells, no significant difference in CRT MFI was found between irradiated and unirradiated groups (Figure 4C). After 12 Gy proton irradiation, HMGB1 expression in Colon-26-shHMGB1 cells still increased significantly. However, compared with the ConshRNA + RT group, HMGB1 expression was significantly reduced in the shHMGB1 + RT group (Figure 4D). This indicated that under radiation treatment, shHMGB1 partially inhibited HMGB1 expression.

Based on the above findings, the CRT and HMGB1 knockdown cell lines were successfully established, and even after high-dose proton irradiation treatment, the expression of DAMPs was still lower than the ConshRNA group. This indicated that the expressions of CRT and HMGB1 had been successfully interfered.

### 3.5 Proton-irradiated tumor cells induce rejection of distal tumor engraftment primarily via HMGB1

**Figure 5:**
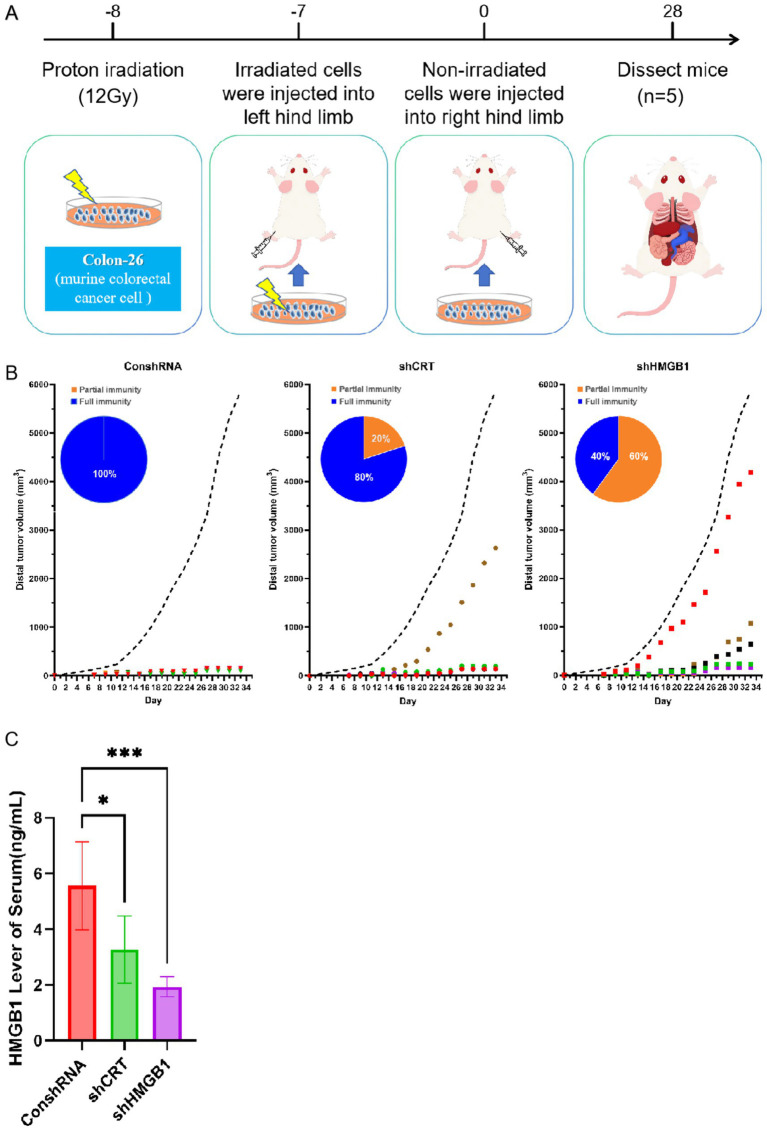
The role of Proton-induced CRT and HMGB1 in distal tumor colonization rejection. **(A)** Experimental schedule for CRT and HMGB1 evaluation. **(B)** Tumor growth after the transplantation of 12 Gy Proton-irradiated cells. The black dotted line indicates mean volume of non-irradiated treated tumor. The blue solid line indicates that the volume of the distal tumor is close to zero, meaning a state of complete immunity. The orange solid line indicates that the volume of the distal tumor is above zero yet remains below those of untreated controls, meaning a state of partial immune control. **(C)** The expression of HMGB1 in serum after the transplantation of 12 Gy Proton-irradiated cells. Ratio of CD4 positive T cell.

To explore the crucial roles of CRT and HMGB1 in the anti-tumor immune response, 5 × 10^5^ Colon-26-shCRT and Colon-26-shHMGB1 cells were exposed to 12 Gy proton beam radiation to make the cells incapable of proliferation while maintaining their antigenic potential. Subsequently, the irradiated cells were implanted into the left hind limb of mice. Seven days later, 1× 10^5^ viable Colon-26 cells were injected into the right hind limb of the mice (Figure 5A). The colonization of distal tumors in mice was visually presented by Figure 5B. Our results showed that compared to the ConshRNA group, CRT knockdown reduced the rejection rate of distal tumor colonization by 20%, while HMGB1 knockdown reduced it by 60%. Interestingly, we observed a significant reduction in serum HMGB1 levels in both the shCRT and shHMGB1 groups compared with the ConshRNA group (Figure 5C). Collectively, these results indicated that proton-induced ICD primarily relied on HMGB1 release rather than CRT membrane translocation to elicit systemic anti-tumor immunity and thereby prevent distal tumor engraftment.

## 4 Discussion

Radiation has long been recognized as an inducer of ICD, and several studies have demonstrated that photon or carbon-ion irradiation can trigger CRT translocation to the plasma membrane and the release of DAMPs such as HMGB1 (23–25). Nevertheless, data on proton beams remain scarce. In this study, we elucidated the mechanism by which proton beam-induced DAMPs inhibited distal tumor colonization and identified the central role of HMGB1 in this process.

Our results showed that high-dose proton irradiation markedly induced HMGB1 release yet exerted no significant effect on CRT membrane exposure. Moreover, *in vivo* experiments revealed that high dose (12 Gy) of proton irradiation significantly elevated serum HMGB1 levels relative to the unirradiated group; concomitantly, CD8 + T-cell activation and IFN-*γ* production were also increased exclusively, implying that the rise in HMGB1 may contribute to the enhanced CD8 + response. These observations underscored that as an important DAMPs, the release of HMGB1 may be involved in regulating the anti-tumor immune response. HMGB1 is a multifunctional regulator that is closely associated with cellular senescence, immune regulation, and inflammatory responses (26). Several researches have proved that HMGB1 can activate pattern recognition receptors such as Toll-like receptors (TLR) and receptor for advanced glycation end products (RAGE), promoting the maturation of DCs and antigen presentation, thereby initiating the immune response mediated by CD8 + T cells (27–32).

Otherwise, using shRNA-mediated knockdown, we observed that depleting HMGB1 markedly reduced the rejection of distal tumor colonazition (by 60%), whereas CRT knockdown produced only a modest 20% decrease. This finding unequivocally demonstrated that HMGB1 release was indispensable for proton-induced rejection of distal tumors. Interestingly, after CRT knockdown, there was a 20% colonization at the distal tumor site, and the level of HMGB1 in the serum also decreased significantly (Figure 5C). This indicated that although CRT was knocked out, the residual distal tumor colonization likely arose through an alternative pathway, such as diminished HMGB1 release. It will be necessary to conduct further research under other proton-beam conditions to clarify the potential auxiliary role of the proton beam-induced CRT.

Despite uncovering the central role of HMGB1, several limitations of this study should be acknowledged. First, the experiments were performed with a fixed 90-MeV proton beam of low linear energy transfer (LET), and CRT translocation was assessed only at 24 h and 48 h. Under these specific conditions, no CRT membrane exposure was detected; however, whether protons of different energies or LET, or at other time points, can trigger CRT externalization remains to be determined. It is necessary to conduct further research covering a wider range of proton energies and LETs and extending the time-course analysis. Second, HMGB1 release may act synergistically with other DAMPs such as ATP and HSP70/90, and the precise molecular mechanisms underlying these interactions remain to be elucidated.

Collectively, this research demonstrates that proton irradiation may elicit a systemic anti-tumor immune response via an HMGB1-dependent pathway, thereby inhibiting distal tumor colonization. These findings not only offer new insights into the immunological mechanisms of proton beam therapy but also provide a theoretical foundation for the development of DAMP-based combinatorial strategies (e.g., proton beam therapy combined with immune-checkpoint inhibitors). Future investigations may focus on delineating the downstream signaling cascades of HMGB1 and its interplay with the tumor microenvironment to further enhance the clinical efficacy of proton beam therapy.

## Data Availability

The original contributions presented in the study are included in the article/supplementary material, further inquiries can be directed to the corresponding author(s).
